# Use of evidence-based pharmacotherapy after myocardial infarction in Estonia

**DOI:** 10.1186/1471-2458-10-358

**Published:** 2010-06-23

**Authors:** Toomas Marandi, Aleksei Baburin, Tiia Ainla

**Affiliations:** 1 Centre of Cardiology, North Estonia Medical Centre Foundation, Tallinn, Estonia; 2 Quality Department, North Estonia Medical Centre Foundation, Tallinn, Estonia; 3 Department of Epidemiology and Biostatistics, National Institute for Health Development; Estonian Centre for Excellence in Behavioural and Health Sciences Tallinn, Estonia; 4 Tampere School of Public Health, University of Tampere, Finland; 5 Department of Cardiology, University of Tartu, Tartu, Estonia

## Abstract

**Background:**

Mortality from cardiovascular disease in Estonia is among the highest in Europe. The reasons for this have not been clearly explained. Also, there are no studies available examining outpatient drug utilization patterns in patients who suffered from acute myocardial infarction (AMI) in Estonia. The objective of the present study was to examine drug utilization in different age and gender groups following AMI in Estonia.

**Methods:**

Patients admitted to hospital with AMI (ICD code I21-I22) during the period of 01.01.2004-31.12.2005 and who survived more than 30 days were followed 365 days from the index episode. Data about reimbursed prescriptions of beta-blockers (BBs), angiotensin converting enzyme inhibitors/angiotensin II receptor blockers (ACE/ARBs) and statins for these patients was obtained from the database of the Estonian Health Insurance Fund. Data were mainly analysed using frequency tables and, where appropriate, the Pearson's χ2 test, the Mann-Whitney U-test and the t-test were used. A logistic regression method was used to investigate the relationship between drug allocation and age and gender. We presented drug utilization data as defined daily dosages (DDD) per life day in four age groups and described proportions of different combinations used in men and women.

**Results:**

Four thousand nine hundred patients were hospitalized due to AMI and 3854 of them (78.7%) were treated by BBs, ACE/ARBs and/or statins. Of the 4025 inpatients who survived more than 30 days, 3799 (94.4%) were treated at least by the one of drug groups studied. Median daily dosages differed significantly between men and women in the age group 60-79 years for BBs and ACE/ARBs, respectively. Various combinations of the drugs studied were not allocated in equal proportions for men and women, although the same combinations were the most frequently used for both genders. The logistic regression analysis adjusted to gender and age revealed that some combinations of drugs were not allocated similarly in different age and gender groups.

**Conclusions:**

Most of the patients were prescribed at least one of commonly recommended drugs. Only 40% of them were treated by combinations of beta-blockers, ACE inhibitors/angiotensin II receptor blockers and statins, which is inconsistent with guideline recommendations in Estonia. Standards of training and quality programs in Estonia should be reviewed and updated aiming to improve an adherence to guidelines of management of acute myocardial infarction in all age and gender groups.

## Background

Beta-blockers (BB), angiotensin-converting enzyme (ACE) inhibitors (or in case of intolerance, angiotensin II receptor blockers (ARBs)) and statins are recommended for most patients after myocardial infarction [1-4]. Underuse of these widely recommended drugs has been previously reported [5,6] despite the fact that efficacy of long-term secondary prevention has been confirmed in many different studies. In addition, non-adherence to beneficial medications may be a marker of other illnesses or habits that result in poor outcomes [7]. Moreover, as well as medication usage (and its success) requiring the coordination of behaviours of both the prescribing physician and the patient, it can be used as a marker to reflect the quality of outpatient care in certain hospitals and/or countries. No studies have been performed to describe outpatient drug utilization patterns in patients who suffered from acute myocardial infarction (AMI) in Estonia. Also, there are no clear factors to explain the very high cardiovascular morbidity and mortality in Estonia. Thus, drug utilization studies in this high risk patient population could have an important input into different strategies for improving the quality of secondary prevention.

The aims of our study were to examine drug utilization in different age and gender groups following acute myocardial infarction in Estonia.

## Methods

Estonian health insurance is a social insurance and it relies on the principle of solidarity. The Estonian Health Insurance Fund (EHIF) covers the cost of health services for 94% of Estonian inhabitants covered by this scheme in case of illness regardless of the amount of social tax paid by the person concerned. From the beginning of the 1990 s the EHIF database has included information about hospitalization and reimbursement of prescriptions for insured persons. The validity of AMI diagnoses has been retrospectively studied - diagnosis of AMI was confirmed in 93.3% of patients in tertiary care hospitals and in 83.5% of patients in secondary care hospitals. Any one of the following criteria satisfied the diagnosis for AMI:

1) typical rise and fall of biochemical markers (troponin T/I, CK-MB/CK-MB mass) and one of the following: a) ischemic symptoms; b) development of pathologic Q waves; c) ECG changes indicative of ischemia; 2) pathologic findings of an AMI by autopsy; 3) the presence of new ST-segment elevation and new chest pain, for those patients who died and for whom no cardiac markers were obtained or cardiac marker(s) were negative because of the short time of attack onset [5].

Beta-blockers, ACE/ARBs and statins can only be purchased by prescription in Estonia, allowing the identification of insured people after the prescription has been reimbursed and the linking of other information included to the database. Estonian Health Insurance Fund database studies do not need Ethics Committee approval in Estonia if personal data that could identify individuals is not included.

For this study the EHIF prepared a list of inpatients treated for AMI (ICD code I21-I22) during the period of 01.01.2004-31.12.2005. This list was then used to identify all prescriptions of BBs, ACE/ARBs and statins reimbursed to these AMI patients during a 12 month period (365 days) after the index episode. Mortality data was also obtained from the Estonian Population Registry for the 12 month period (365 days) after the index episode. The index episode was defined as the first hospitalization due to AMI. Data were made available to us such that individuals could not be identified and additional analyses were performed.

Prescriptions for long-term treatment in Estonia are provided as a set of three prescriptions for two months and can be purchased at the time or after every two months - thus we decided to analyse drug utilization annually. Drug utilization was analysed by the use of the defined daily dosages (DDD) concept [8]. The DDD is the assumed average maintenance dose per day for a drug used for its main indication in adults. The purpose of the DDD system is to serve as a tool for drug utilization research in order to improve quality of drug use. The total amount of drugs from each drug group reimbursed during the study period was presented as DDD to minimize confusion from different drugs and doses. This total was divided by life days per 12 months (365 days) of follow-up period to demonstrate drug utilization. Medicines reimbursed and DDDs used in calculations were as follows: metoprolol 150 mg, bisoprolol 10 mg, atenolol 75 mg, nebivolol 5 mg, captopril 50 mg, enalapril 10 mg, lisinopril 10 mg, perindopril 4 mg, ramipril 2.5 mg, quinapril 15 mg, fosinopril 15 mg, zofenopril 30 mg, imidapril 10 mg, losartan 50 mg, eprosartan 600 mg, valsartan 80 mg, irbesartan 150 mg, candesartan 8 mg, telmisartan 40 mg, olmesartan 20 mg, simvastatin 15 mg, pravastatin 20 mg, fluvastatin 40 mg, atorvastatin 10 mg, rosuvastatin 10 mg [8]. At least one reimbursed prescription for a particular drug group was needed to define a patient as a particular drug group user or treated patient. All drugs reimbursed were considered as used by the patient.

Data from patients who survived more than 30 days after the index episode were used to determine drug utilization.

### Statistical analysis

Data were mainly analysed using frequency tables. For descriptive statistics, the results were given as mean values with the standard deviation, and for the analysis of treatment utilization, median dosages with 95% confidence intervals were calculated for selected age groups. Categorical data were compared with Pearson's χ2 test, continuous data with the t-test and median dosages were compared with the Mann-Whitney U-test. A logistic regression method was used to investigate the relationship between drug allocation and age and gender with individual drugs and drug combinations used as dependent variables. Results were given as odds ratios and their 95% confidence intervals. Analysis was performed with statistical software package Stata 11 (Stata, College Station, TX, USA).

## Results

During the study period, 4900 patients were hospitalized due to AMI (Table 1). Men comprised more than half of the patients. The combined mean age (± SD) for men and women was 69 years (± 11.5). The mean age of the men was significantly lower than for the women. Eight hundred seventy five patients, 407 men (mean age ± SD, 71.06 ± 11.5 years) and 468 women (mean age ± SD, 76.95 ± 8.73 years) died within 30 days from index episode.

**Table 1:** Characteristics of all inpatients with acute myocardial infarction in Estonia (n = 4900).

	Men	Women
Total number of patients, (%)	2772 (56.6)	2128 (43.4)**
Number of deaths, (%)	709 (25.6)	744 (35)**
Number of treated patients, (%)	2260 (81.5)	1594 (74.9)**
Age (years; mean ± SD)	65.66 ± 11.56	73.61 ± 9.81**
20-39 years, No. (%)	36 (1.30)	4 (0.19)**
40-59 years, No. (%)	786 (28.35)	183 (8.60)**
60-79 years, No. (%)	1666 (60.10)	1333 (62.64)
80-99 years, No. (%)	284 (10.25)	608 (28.57)**

**P < 0.01 for comparison between men and women. Age was compared with the t-test, otherwise Pearson's χ2 test was used.

Closer inspection of the age distribution revealed that there was a higher proportion of men in the 20-59 years age group while women were more prevalent in the 80-99 years age group. The age group of 60-79 years included quite similar proportions of men and women. Hence, among those who survived more than 30 days, more patients in the younger age groups were men than women. The proportion of treated patients was significantly different in men and women but this distinction disappeared after exclusion of patients who died within the first 30 days (Table 2).

**Table 2:** Characteristics of patients who survived more than 30 days (n = 4025).

	Men	Women
Total number, (%)	2365 (85.3)	1660 (78.0)**
Number of deaths, (%)	302 (12.8)	276 (16.6)**
Number of treated patients, (%)	2235 (94.5)	1564 (94.2)
Age (years; mean ± SD)	64.73 ± 11.53	72.66 ± 9.90**
20-39 years, No. (%)	34 (1.44)	4 (0.24)**
40-59 years, No. (%)	740 (31.29)	166 (10.00)**
60-79 years, No. (%)	1383 (58.48)	1075 (64.76)**
80-99 years, No. (%)	208 (8.79)	415 (25.00)**

**P < 0.01 for comparison between men and women.

Age was compared with the t-test, otherwise Pearson's χ^2 ^test was used.

### Drug utilization

Of the 4900 inpatients, 3854 (78.7%) were treated by at least one of BBs, ACE/ARBs and/or statins, whilst no reimbursed prescriptions were found in the Estonian Health Insurance Fund database for 1046 (21.3%) patients during the 365 days after the index episode.

Of the 4025 inpatients who survived more than 30 days, 3799 (94.4%) were treated by at least one of BBs, ACE/ARBs and/or statins, whilst no reimbursed prescriptions were found in the Estonian Health Insurance Fund database for 226 (5.6%) patients.

**Figure 1:**
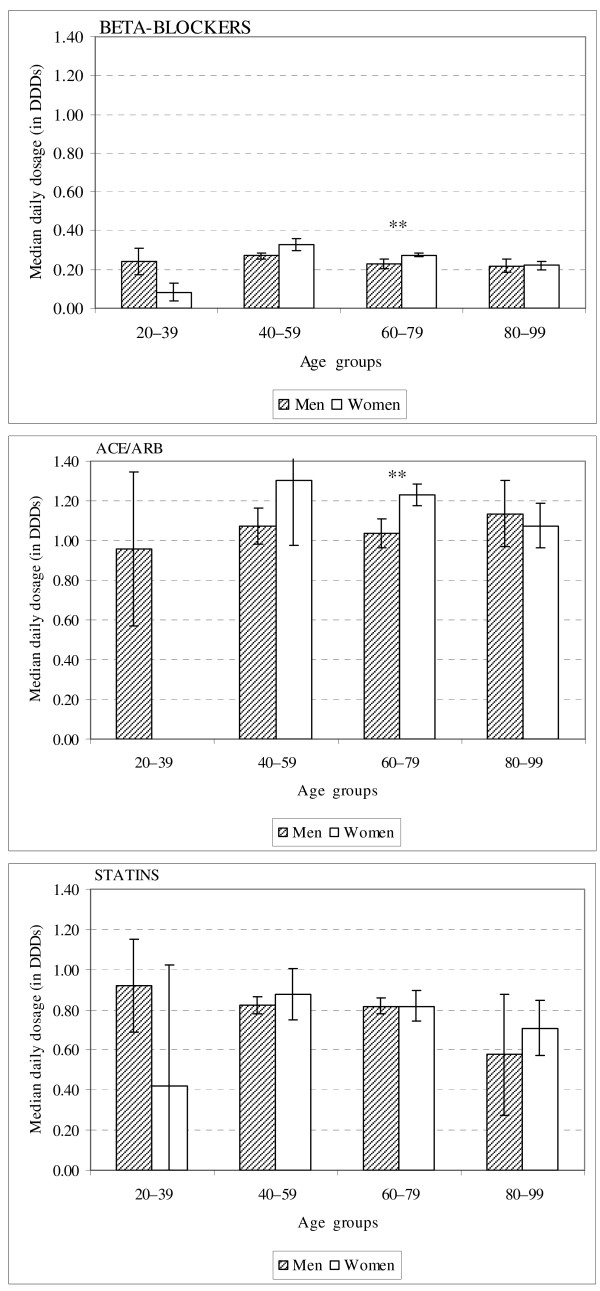
**Median daily dosages of the treatments in age groups for men and women who survived more than 30 days (n = 4025)**. Median daily dosages are presented as DDDs with 95% confidence intervals. DDD, defined daily dosages; ACE/ARB, angiotensin converting enzyme inhibitors/angiotensin II receptor blockers. DDDs used were as follows - metoprolol 150 mg, bisoprolol 10 mg, atenolol 75 mg, nebivolol 5 mg, captopril 50 mg, enalapril 10 mg, lisinopril 10 mg, perindopril 4 mg, ramipril 2.5 mg, quinapril 15 mg, fosinopril 15 mg, zofenopril 30 mg, imidapril 10 mg, losartan 50 mg, eprosartan 600 mg, valsartan 80 mg, irbesartan 150 mg, candesartan 8 mg, telmisartan 40 mg, olmesartan 20 mg, simvastatin 15 mg, pravastatin 20 mg, fluvastatin 40 mg, atorvastatin 10 mg, rosuvastatin 10 mg [8]. **P < 0.01 for comparison between men and women with Mann-Whitney U-test

Median daily dosages of BBs, ACE/ARBs and statins are presented as DDDs with 95% confidence intervals for age groups and gender in Figure 1. Median dosages of these medicines were very similar in different age groups, except in case of BBs for women, but this was due to the small number of women in the age group of 20-39 years. Comparison between men and women revealed that the median daily dosage differed significantly in the age group of 60-79 years. However, median BB dosages allocated were commonly less than 0.4 DDD per day. A similar difference was observed for ACE/ARB and also in age group of 60-79 years, but the median daily dosages were much higher and mostly exceeded 1 DDD per day. Statins were allocated similarly for both genders and all age groups.

The number of patients who were treated by various combinations of drugs is given in Table 3. Pearson's χ^2 ^test indicated that the treatments were not allocated in equal proportions for men and women, although the same combinations were most frequently used for both genders. The results of logistic regression analysis of allocated drug combinations adjusted to gender and age are presented in Table 4. This analysis revealed that some combinations of drugs were not allocated similarly in different age and gender groups.

**Table 3:** Combinations of prescribed treatments for men and women who survived more than 30 days (n = 4025).

Treatment combinations	Men (%)	Women (%)
BB, No. (%)	176 (7.44)	123 (7.41)
ACE/ARB, No. (%)	170 (7.19)	145 (8.73)
Statins, No. (%)	17 (0.72)	12 (0.72)
BB & ACE/ARB, No. (%)	602 (25.45)	525 (31.63)**
BB & statins, No. (%)	130 (5.50)	49 (2.95)**
ACE/ARB and statins, No. (%)	141 (5.96)	63 (3.80)**
BB & ACE/ARB & statins, No. (%)	999 (42.24)	647 (38.98)
No drugs, No. (%)	130 (5.50)	96 (5.78)
Total, No. (%)	2365 (100.00)	1660 (100.00)

BB, beta-blockers; ACE/ARB, angiotensin converting enzyme inhibitors/angiotensin II receptor blockers.

**P < 0.01 for comparison between men and women with Pearson's χ^2 ^test.

**Table 4:** Relationship between drug allocation and age and gender in patients who survived more than 30 days.

Drug	Male OR (95% CI)	Female OR (95% CI)	20-39 years OR (95% CI)	40-59 years OR (95% CI)	60-79 years OR (95% CI)	80-99 years OR (95% CI)
BB	1.00	0.79 (0.61-1.02)	1.00	0.56 (0.17-1.91)	0.97 (0.29-3.19)	1.93 (0.58-6.47)

ACE/ARB	1.00	0.89 (0.70-1.14)		1.00	2.49 (1.67-3.70)**	5.69 (3.66-8.82)**

Statins	1.00	1.19 (0.55-2.57)		1.00	0.88 (0.38-2.06)	0.17 (0.02-1.37)

BB & ACE/ARB	1.00	1.15 (0.99-1.33)	1.00	0.79 (0.37-1.70)	1.21 (0.57-2.57)	1.92 (0.89-4.14)

BB & statins	1.00	0.68 (0.49-0.92)**	1.00	0.66 (0.19-2.21)	0.81 (0.24-2.66)	0.28 (0.08-1.06)

ACE/ARB & statins	1.00	0.63 (0.44-0.89)**	1.00	0.48 (0.18-1.28)	0.32 (0.12-0.84)**	0.19 (0.06-0.58)**

BB & ACE/ARB & statins	1.00	1.21 (1.05-1.39)**	1.00	1.94 (1.00-3.77)	1.02 (0.53-1.96)	0.28 (0.14-0.55)**

**P < 0.05 by logistic regression method.

BB, beta-blockers; ACE/ARB, angiotensin converting enzyme inhibitors/angiotensin II receptor blockers; OR, Odds ratio; CI, confidence interval

## Discussion

This study was the first one aimed at examining long-term drug utilization in different age and gender groups following acute myocardial infarction in Estonia. It was found that 94.4% of patients admitted to hospital due to AMI and who survived more than 30 days were reimbursed at least one prescription of the widely recommended beta-blockers, ACE inhibitors/ARBs or statins. The proportion of treated patients was very similar for men and women, although logistic regression analysis adjusted for gender and age revealed that some combinations of drugs were not allocated similarly in different age and gender groups. However, it should be emphasized, that only 40% of treated patients used medicines from all of the studied groups during the year. Beta-blockers were commonly used in moderate doses (the most common was metoprolol, daily dose 50 mg). This finding is very similar to those from earlier studies where differences between routine clinical practice and recommendations were reported [6,9-12]. Differences between the characteristics of patients included in clinical trials and treated in everyday practice was suggested as being the main reason for these differences and external validity of data from clinical trials has been debated [13]. Compared to Estonian data from 2001 where treatment recommendations at discharge were reported [5], notable improvements in drug usage were observed; among patients who survived more than 30 days after hospitalization due to acute myocardial infarction, more patients used widely recommended drugs. This could be a result of training programs being provided to physicians during the time between the two studies, and of the implementation of treatment guidelines in clinical practice. However, the number of patients being prescribed these drugs is still not consistent with guideline recommendations and needs further improvement.

A small proportion of patients were not using any recommended medicines during the study period. In addition to the expected lack of effect from medicines, other factors could have influenced this finding, such as the age of the patients, adverse events during the acute attack and concomitant diseases. For example, the characteristics of patients who died during the first 30 days after the attack are different from those of the total population and the population who survived more than 30 days - this group consisted of more women and elderly patients.

The Estonian Myocardial Infarction Registry data showed 30-day mortality in 2001-2002 as 13.2% in men and 17.4% in women [14]. The results from our study indicated that a high level of mortality was still occurring after acute myocardial infarction in Estonia, despite treatment improvements - 14.7% of men and 22% of women die during a 30-day period and altogether 25.6% of men and 35% of women die during the year after an acute attack. Mortality rates reported elsewhere varied widely [6,15-17]. A positive relationship between adherence to evidence-based pharmacotherapy and survival following AMI has been reported [18,19]. To date, several risk scores have been suggested to aid treatment decisions. For example, the GRACE risk score [20] was suggested for the determination of patients with a high risk of death and AMI, and to help physicians optimize revascularization decisions [21]. Although patient risk profiles can be among the factors determining treatment decisions and outcomes [15], the routine scoring of AMI patients is not common in Estonia. Moreover, there is no evidence of specific genetic, socioeconomic or other factors which could explain the high mortality rates after AMI in Estonia. According to recent findings from unpublished data by Blöndal M *et al*, improved access to percutaneous coronary interventions during the last few years did not positively influence the annual mortality rate of patients who suffered from acute myocardial infarction.

There were a number of limitations to this study. Firstly, the database that was used does not record data about patients who are not covered by the Health Insurance Fund Scheme or prescriptions that are not reimbursed by the Estonian Health Insurance Fund. Thus, we did not have data about the medical care of 6% of Estonian inhabitants or data about the use of medicines not reimbursed. However, the proportion of patients and prescriptions that was not included was too small to affect the results of this study. Databases of reimbursed prescriptions have been used in drug utilization studies elsewhere and were considered to adequately represent real situations.

Secondly, the DDDs that were used can be difficult to interpret in terms of clinical efficacy. However, as patients were treated with many different medicines - 4 beta-blockers, 16 ACE/ARBs, 5 statins - it was decided that the DDD was the simplest way to present drug utilization and to compare drug utilization patterns in different age and gender groups.

Thirdly, data about treatment during hospital stays (e.g. revascularization methods used), concomitant diseases and other medicines used (e.g. clopidogrel, antiinflammatory drugs, aspirin) was not collected and analysed due to different reasons. For example, aspirin and some anti-inflammatory drugs are defined as over-the-counter (OTC) drugs not included in the Estonian Health Insurance Database. No data on the use of these drugs by post-AMI patients can be obtained from pharmacies either. Clopidogrel was not recommended to all AMI patients during the study period and had limited use due to a restricted reimbursement policy. Thus, we decided not to include data about clopidogrel use in our study. Reporting of concomitant diseases in the Estonian Health Insurance Fund database is not validated and would also be misinterpreted due to the implemented financing model that was partly based on disease-related group methodology.

Finally, our data contained no information about adverse events and intolerances, which could explain our findings of quite low median daily doses in different drug groups.

## Conclusions

Most of patients who survived 30 days after acute myocardial infarction were prescribed at least one of commonly recommended drugs. Only 40% of these patients were treated with a triple combination of beta-blockers, ACE inhibitors/angiotensin II receptor blockers and statins, which is inconsistent with the recommendations of guidelines in Estonia. Standards of training and quality programs in Estonia should be reviewed and updated aiming to improve an adherence to guidelines of management of acute myocardial infarction in all age and gender groups.

## Competing interests

The authors declare that they have no competing interests.

## Authors' contributions

TM, AB and TA participated in the design of the study and in writing the manuscript and AB performed the statistical analyses. All authors read and approved the final manuscript.

## Authors' information

TM is a president of the Estonian Society of Cardiology and a member of the Scientific Board of Estonian Myocardial Infarction Registry.

TA is a member of the board of the Estonian Society of Cardiology and a head of the Scientific Board of Estonian Myocardial Infarction Registry.

## Pre-publication history

The pre-publication history for this paper can be accessed here:

http://www.biomedcentral.com/1471-2458/10/358/prepub
